# Response of a *Chloroidium saccharophilum* Strain to Extreme Conditions of the Atacama Desert

**DOI:** 10.3390/biology15090698

**Published:** 2026-04-29

**Authors:** Nicolás Lobos, Diego Igor, Nelson Cepeda, Lía Ramirez, Juan Pablo Díaz

**Affiliations:** 1 Faculty of Renewable Natural Resources, Arturo Prat University, Iquique 1100000, Chile; nilobosg@estudiantesunap.cl (N.L.);; 2 Núcleo de Investigación Aplicada e Innovación en Ciencias Biológicas, Facultad de Recursos Naturales Renovables, Iquique 1110939, Chile

**Keywords:** *Chloroidium saccharophilum*, carotenoids, extremophile, Mars, microalgae

## Abstract

*Chloroidium saccharophilum* is a resilient green microalga with a broad ecological distribution and an increasing biotechnological interest due to its tolerance of extreme environmental conditions. In this study, a sample of *C. saccharophilum* from the Laguna Blanca aquifer (Magallanes, southern Chile) was physiologically and phylogenetically characterized. This is the first confirmed evidence of this strain in the Southern Cone. Molecular identification based on ITS rDNA sequencing and ITS2 secondary structure analysis confirmed its taxonomic location, showing high similarity with reference strains and no compensatory base changes. Growth performance was analyzed under controlled laboratory conditions and under outdoor desert cultivation in the Atacama Desert, focusing on temperature, salinity, nutrients limitation, and high solar irradiance operational conditions. The strain exhibited optimal growth at 22 °C under laboratory conditions and demonstrated a strong tolerance to high salinity (150 g L^−1^ NaCl). Outdoor raceways cultivation revealed a negative relationship between temperatures above 25 °C and biomass accumulation, while nutrients depletion and strong irradiance caused moderate carotenoid accumulation. However, the low amount of carotenoid yields remained constant, even under combined stress conditions. In general, the results highlight the ecological adaptability and the stress tolerance of *C. saccharophilum*, supporting its potential application in saline bioprocesses and bioremediation. Nevertheless, the limited production of carotenoid synthesis suggests that additional or combined stress strategies will be required to enhance the production of high-value metabolites. This study expands the biogeographical knowledge of *C. saccharophilum* and provides a physiological baseline for future optimization studies in extreme and Mars-analog environments.

## Simple Summary

Microalgae are tiny organisms that play an essential role in nature, especially in places with harsh environmental conditions. In this study, we examined a microalga called *Chloroidium saccharophilum*, which we found in Laguna Blanca, Chile. This is important because this species had never been reported in Chile before or in this region of South America. Its presence in such an extreme environment makes it valuable for scientific and biotechnological research. This microalga is naturally resistant to very salty water, strong sunlight, and temperature changes—conditions that would harm many other organisms. These special abilities give it potential as a natural source of useful compounds such as antioxidants. Antioxidants help protect cells from damage caused by stress and inflammation, which is relevant for health-related products and other applications. Although the microalga showed strong growth, it produced only small amounts of antioxidants under the conditions we tested. This suggests that more research is needed to discover the best environmental factors that encourage higher production of these beneficial compounds. Learning about organisms that thrive in extreme areas can support new sustainable technologies and contribute to economic and social development in remote regions.

## 1. Introduction

Microalgae are worldwide microorganisms found in all aquatic environments where sunlight and carbon dioxide are available, and oxygen can be released as a subproduct of photosynthesis [1]. They are largely limited to aquatic habitats due to their lack of mechanisms to withstand desiccation. Nevertheless, their remarkable omnipresence arises from a wide range of physiological and biochemical adaptations that allows them to specialize, survive, and succeed in different environments in contrast to other organisms that have significant limitations. As a result, microalgae can adapt to extreme conditions of temperature, pH, pressure, and salinity [2].

In this context, the Atacama Desert in northern Chile is characterized as one of the most arid and oldest deserts on Earth. Research on its microbial ecosystems was initially promoted by NASA decades ago in the framework of astrobiology. It was intensified after 2003, when the Atacama Desert was recognized as a terrestrial analog of Mars [2]. Since then, a variety of ecological niches, including hazardous environments such as quartz rocks, Andean fumaroles, halite evaporites, and coastal caves, have been explored, revealing the remarkable ability of microbial life to adapt to extreme conditions such as severe water scarcity, high salinity, and intense ultraviolet radiation [3]. For instance, previous studies developed in volcanic gypcrete deposits of the Atacama Desert have investigated endolithic phototrophic microorganisms. Using Raman spectroscopy, carotenoid pigments were mapped, revealing pigment gradients that are essential for the survival of algae and cyanobacteria under extreme environmental stress. Such is the case of *C. saccharophilum*, a species that was previously classified within the genus Chlorella [4,5]. Species traditionally identified as Chlorella have attracted considerable scientific interest due to their versatility in the production of dietary supplements, functional foods, and nutraceuticals. These properties not only emphasize their current relevance but also suggest an outstanding role related to the future direction of the food industry [3].

Microalgal growth and biochemical composition are critical factors in determining their potential applications. In microalgae, these characteristics are strongly influenced by cultivation parameters such as pH, salinity, carbon source and concentration, light intensity, and nutrients limitation [5,6]. Consequently, laboratory-based studies are essential for optimizing these parameters to maximize biomass production or the accumulation of specific compounds for industrial applications [6]. Furthermore, the dynamics of the chlorophyll (Chl) a to b ratio in microalgae are intrinsically linked to their interconversion through the chlorophyll cycle, their distinct roles in the photosynthetic process, and their capacity for acclimation to environmental variability. Understanding this relationship is essential, as it directly influences into growth optimization and productivity, which are highly important for biotechnological applications and environmental monitoring [5,7,8,9,10,11].

*C. saccharophilum* (W.Krüger) Darienko is a particularly resilient microalga, capable of enduring adverse environmental conditions, including high salinity and high concentrations of heavy metals, which it bioaccumulate, as well as low temperatures and limited nutrient availability [5,10]. Initially misidentified as *Chlorella vulgaris* (Beijerinck 1890) [12], *C. saccharophilum* is a worldwide green microalga with an ellipsoidal morphology and few distinctive morphological traits. Like other microalgae, it can synthesize a large range of carotenoids, including xanthin, violaxanthin, neoxanthin, α-carotene, β-carotene, and lutein, among others [13].

The characterization of microalgal strains is fundamental in biotechnology due to their capacity to biosynthesize metabolites with different applications. In particular, the production of antioxidants, including pigments, has gained significant interest due to their potential for human consumption and their ability to enhance the organoleptic properties of various products. Accordingly, the present study aimed to characterize the response of the *C. saccharophilum* strain to the extreme environmental conditions of the Atacama Desert, specifically in the “Pampa del Tamarugal”, using an inoculum originally isolated from the Laguna Blanca aquifer in the Magallanes Region, southern Chile. Additionally, its potential relevance as a Mars-like environmental analog within the Atacama Desert was analyzed. Previous results have indicated that *Chlorella vulgaris* (Baker strain) might be used as a raw material for biodiesel production [14], whereas *C. saccharophilum* (Coliumo strain) seems to be more appropriate for animal nutrition. However, it is important to highlight that cultivation conditions were not optimized in that study, and further optimization could significantly and differentially increase total lipid productivity and improve lipid quality for fuel production and/or animal feed applications [4].

## 2. Materials and Methods

### 2.1. Sample Collection and Strain Isolation

**Figure 1:**
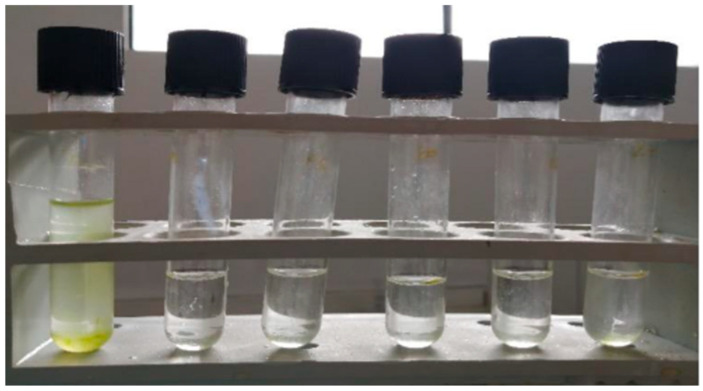
An axenic monoalgal culture of *C. saccharophilum*. The first tube on the left corresponds to the stock culture, while the remaining tubes represent monoalgal cultures.

A water sample containing populations of *C. saccharophilum* was collected from the Laguna Blanca aquifer (52.47991° S, 71.25183° W), located approximately 3 km from Route 9 North in the Magallanes Region, southern Chile, in March 2022. The Laguna Blanca aquifer is situated in a cold steppe climate characterized by noticeable variability regarding rainfall. Although the aquifer was not previously disrupted, the aquifer has now been affected by livestock activities. It supports wetlands and unique soils, which are characterized by low mean temperatures (approximately 7 °C), fluctuating precipitation levels (233–932 mm year^−1^), and saline soils rich in organic matter (histosols). The water exhibits slight salinity, influenced by climate change and the potential accumulation of salts. Nevertheless, previous studies indicate that the aquifer is resilient, maintaining stable water levels due to a balance between recharge processes, even under changing climatic conditions [15]. The sample was placed in a 1.5 mL Eppendorf tube and relocated at the laboratory of microalgae biotechnology, Faculty of Renewable Natural Resources, Arturo Prat University, Iquique, Chile. During the initial stage, the sample was cultured in KSP medium [16] (Table 1). The strain was isolated using monoclonal culture techniques (Figure 1). A 10 mL inoculum was centrifuged at 1789× *g* for 10 min, and the resulting pellet was resuspended in 10 mL of modified KSP medium. Individual microalgal cells were isolated through serial dilutions to obtain approximately one cell per 10 µL. The appearance of a single cell was verified under a microscope, and the cell was inoculated into a test tube containing 3 mL of modified KSP culture medium. The cultures were kept at room temperature in a location protected from direct sunlight and were incubated for an undefined period of time until visible coloration developed.

### 2.2. Morphological and Phylogenetic Characterization and ITS2 Secondary Structure Analysis

For the visual observation of *C. saccharophilum* morphological characteristics, a Carl Zeiss Axiostar Plus optical microscope (ZEISS, Oberkochen, Germany) with 60× magnification was used. Images of focused cells were captured and processed using ImageJ software v. 1.54b 2023 (NIH, Bethesda, MD, USA) [17]. The observed morphological features were compared with the reference data available in the AlgaeBase database (www.algaebase.com). The microscope used for confocal microscopy was a Zeiss 710 laser scanning.

For the identification of the species and phylogenetic analysis, *C. saccharophilum* samples were collected, and cells were isolated and washed with deionized water to remove the high NaCl content of the culture medium. Genomic DNA was extracted, and sequencing of the ITS rRNA gene marker was performed using primers ITS4 and ITS5. Additionally, the 18S rRNA gene marker was amplified using primers Tar18S_F and Tar18S_R. Sequencing was carried out using an ABI 3500 capillary sequencer at the AUSTRAL-omics Core Facility, University Austral of Chile (Valdivia, Chile). Sequences were manually edited by visual inspection of chromatograms and assembled into consensus sequences using BioEdit^®^ version 7.7 (North Carolina State University/University of South Carolina School of Medicine. North Carolina/South Carolina, USA) [18]. Closely related sequences were retrieved from the BLASTn v 2.16.0 database [19] for phylogenetic analysis. Multiple sequence alignment was performed in MEGA X using the MUSCLE algorithm [20,21]. Phylogenetic trees were inferred using the maximum likelihood method with 1000 bootstrap replicates, allowing comparison with other *C. saccharophilum* strains. The obtained sequences were deposited in the European Nucleotide Archive (ENA) under the study accession number PRJEB96322.

Using the consensus sequence, the regions amplified by primers ITS4 and ITS5, specifically the partial 18S rRNA, ITS1, 5.8S rRNA, and ITS2 and partial 28S rRNA genes, were annotated using the ITS2 Database [22,23]. The extracted ITS2 sequence was imported into the RNAfold web server to predict its secondary structure with the default parameters. To analyze the presence of compensatory base changes (CBCs) and compare the query sequence with a reference (PX279531), the secondary structure in Vienna format was imported into the CBCAnalyzer software Version 1.0.3 [24].

### 2.3. Culture Conditions

The culture medium used was KSP (Table 1) [15]. Cultures were maintained under an irradiance of 200 µmol m^−2^ s^−1^ (LED Greenhouse lighting, Company MEAN WELL Mansfield, TX, USA, Model HLG-240 H), with a 14/10 h light–dark photoperiod and continuous aeration at 4 L min^−1^ without CO_2_ supplementation. Cultures were scaled up whenever the cell concentration reached 4.0 × 10^5^ cells mL^−1^, as growth rates decreased beyond this density. The process began with a *C. saccharophilum* stock culture inoculated into 100 mL of KSP medium under the specified irradiance conditions. Once the target cell concentration was achieved, aeration was applied at a flow rate of 4 L min^−1^ to preserve the environmental conditions. Subsequently, the biomass was transferred to a pilot-scale raceway pond using a 10 L inoculum with a specific cell concentration, where growth behavior and pigment accumulation capacity under environmental stress conditions were analyzed.

#### 2.3.1. Performance Under Laboratory Conditions

To determine the most suitable temperature for growth, a factorial experimental design was conducted with temperature and replications as factors, consisting of three treatments and three replicates per treatment. The evaluated temperatures were 16 °C, 19 °C, and 22 °C. These treatments were based on modifications of the experimental conditions reported by Wu et al. (2016) [25]. Each replicate consisted of 3 L of KSP medium with an initial cell concentration of approximately 6.3 × 10^4^ cells mL^−1^. Cultures were grown for 15 days. Daily cell counts were performed using a Neubauer chamber, with ten counts conducted per replicate and treatment. The average value was calculated and used to estimate the cell concentration using (Equation (1)). To characterize the strain performance, several parameters were measured over time, including nutrients consumption, growth rate, pH, dissolved oxygen concentration, and the effect of temperature.

Cell/µL = (N° of counted cells/Volume) ∗ Dilution factor(1)

#### 2.3.2. Performance Under Outdoor Conditions

For outdoor cultivation, *C. saccharophilum* cultures were initially adapted to desert conditions at the Canchones Experimental Station, Arturo Prat University, located in the Atacama Desert (Tarapacá, Chile). Following acclimation, cultures were scaled up, and the same variables described above were monitored, comparing growth rates across temperature treatments. In this stage, cultures were kept for 30 days to analyze the effects of room temperature. Subsequently, outdoor cultivation was conducted at Solarium Biotechnology S.A., located at kilometer 15 of Route A-665, Pozo Almonte, Chile. Pilot-scale raceway-type bioreactors were used, measuring 1.5 m in length and 0.5 m in width, with a 60 L working volume and a mixing speed of 12 rpm. An experimental design based on nutrients restriction was implemented, specifically reducing nitrate concentrations in the original KSP medium, with the following treatments: T1, 10% of the original nitrate concentration; T2, 15%; and T3, 0%. This strategy was applied to induce carotenoid accumulation through solar stress, increased salinity, and nutrients deprivation [25,26,27]. Each treatment received 20 L of *C. saccharophilum* stock culture previously grown at the Canchones Experimental Station under a maximum irradiance of 2277 µmol m^−2^ s^−1^.

Daily irradiance was measured using a Delta OHM HD2302.0 light meter (Unisource Ingeniería Ltda, Santiago de Chile, Chile) with a 14/10 h photoperiod. The experiment persisted for 15 days, at which cell growth and minimum and maximum temperatures were recorded.

### 2.4. Cell Growth and Biomass Determination

Both indoor and outdoor experiments were conducted. Laboratory assays allowed the determination of optimal growth and development conditions for the strain. Temperature, cell growth over time, nitrate and phosphate consumption, oxygen production, pH, and cell size were monitored. Daily measurements of these variables were noted along the 15-day period. Temperature experiments were performed using three treatments and three replicates at T1 16 °C, T2 19 °C, and T3 22 °C [28] (Table 2). Cell growth was quantified using a Neubauer chamber with a depth of 0.01 mm and a manual counter. Ten counts were performed per sample, and the average value was used to estimate cell concentration (cells mL^−1^). Nitrate and phosphate consumption were determined using colorimetric and spectrophotometric methods following Hannah Instruments protocols, using a Visible Iris Spectrophotometer (HI801) at wavelengths of 480 nm and 610 nm, respectively. Dissolved oxygen concentration was measured using a YSI Model 55/25 dissolved oxygen meter (Antumec Ingenieria Y Servicios LTDA, Santiago de Chile, Chile) while pH was measured using a YSI Model WQ201 sensor (Antumec Ingenieria Y Servicios LTDA, Santiago de Chile, Chile). Cell size was determined by analyzing micrographs with ImageJ software, which allowed measurement of cell diameter and cell number.

To calculate the growth rate, the biomass concentration was required. For this purpose, a calibration curve was generated to establish the relationship between dry cell concentration (CC) and absorbance. Six different concentrations of *C. saccharophilum* were prepared following the methodology described by Kazeem et al., 2018 [29]. These concentrations were obtained by serial dilution, resulting in approximate cell densities of 5000, 10,000, 15,000, 20,000, 25,000, and 30,000 cells mL^−1^. For each concentration, 100 mL of stock culture was collected, followed by cell counting and absorbance measurements. Subsequently, the samples were filtered using a vacuum pump and a 0.45 µm pore size filter ADVANTEC GC50, 47 mm diameter (Equilab Ltda, Santiago de Chile, Chile), which had been previously weighed. After the initial filter weight, filtration was performed to retain *C. saccharophilum* cells, sodium chloride, and residual culture medium on the filter. The filter was dried in an oven at 80 °C for 6 h to evaporate the water content. After drying, the filter was weighed again, now containing *C. saccharophilum* cells and sodium chloride, allowing the determination of dry cell weight. Finally, the filter was incinerated in a muffle furnace for 8 h to combust the algal biomass. Dry biomass was obtained by difference, considering the remaining ash in the filter. Dry weight values were correlated with absorbance and CC, producing a calibration curve that enabled the conversion of absorbance to CC and biomass concentration (CB) as well as the conversion of CC to CB. This calibration curve was used to calculate the biomass productivity (Equation (2)) and the growth rate (Equation (3)).

(2) PB=(CBf−CB0)/(tf−t0)

(3) µ=(ln(CBf/CB0)−ln(CBn/CB0))/(tf−t0)

where

PB = biomass productivity (g L^−1^ day^−1^).
µ = specific growth rate (day^−1^).
CBf = final biomass concentration (g L^−1^).
CB0 = initial biomass concentration (g L^−1^).
tf = final time (days).
t0 = initial time (days).

### 2.5. Nitrate and Phosphate Consumption and Oxygen Production

Nutrients concentrations were measured using colorimetric techniques established by Hanna Instruments. Nitrate concentration was measured using a nitrite meter (model HI93707), which provides readings in the range of 0–600 ppm NO_2_–N. An initial nitrate concentration of 500 ppm was recorded on day 1, and measurements were taken daily throughout the acclimation period. Phosphate concentration was measured using a phosphate meter (model HI93713), with a measurement range of 0–100 ppm PO_4_^3−^. An initial concentration of 50 ppm was recorded, and daily measurements were taken during the experimental period. Consumption of both nutrients, i.e., daily nitrate and phosphate, was determined based on the decrease in nutrient concentration relative to the initial value. pH was measured daily using a Hanna Checker HI98103 pH meter (Hannah instruments, Santiago de Chile, Chile), while dissolved oxygen concentration was measured using a YSI 55/25 model dissolved oxygen meter (Antumec Ingenieria Y Servicios LTDA, Santiago de Chile, Chile).

### 2.6. Pigment Determination

Chlorophyll a, b, and total carotenoid concentrations in dry samples were determined using spectrophotometric methods according to Tavakoli et al. [30]. Pigment extracts were prepared from dry samples at a concentration of 1 mg mL^−1^ using 70% (*v*/*v*) ethanol. Absorbance was measured at 470, 647, and 664 nm. Chlorophyll concentrations were calculated using the following equations.

(4) $${Chl}_{a}=11.93*A_{664}-1.93*A_{647}$$

(5) $${Chl}_{b}=20.36*A_{647}-5.50*A_{644}$$

(6) $$Carotenoids=7.6*(A_{470}-1.49*A_{510})$$

For carotenoid quantification, we used the protocol described by Arredondo et al. [31] for green algae. The following procedure was used: three samples were collected daily from each *C. saccharophilum* treatment. From each sample, 4 mL were transferred to Eppendorf tubes and centrifuged at 6000 rpm for 15 min. The supernatant was discarded, and the pellet was washed with double-distilled water to remove salt waste. Then, the volume was adjusted to 3 mL with 90% acetone, and samples were kept on ice for 24 h. After extraction, samples were centrifuged again in 1789× *g* for 15 min. Pigment quantification was performed using a UV–vis spectrophotometer Hanon Instruments, model I3 (Jinan, China) measuring absorbance at 664, 647, 510, and 470 nm, corresponding to Chl a, b, and carotenoids, respectively. Carotenoid concentration was calculated using Arredondo’s equation for green algae and expressed as pg cell^−1^.

### 2.7. Software and Statistical Analysis

Data were processed using Microsoft Excel 2016^®^ and RStudio^®^ (64-bit version 4.2.2). Model performance was developed using the coefficient of determination (R^2^). Statistical analyses were conducted in RStudio^®^ (Posit, PBC, Boston, MA, USA), including tests for normality, homogeneity of variances, and significance. For normally distributed data, analysis of variance (ANOVA) followed by Duncan’s multiple range test was applied. For non-parametric data, a Kruskal–Wallis test was performed.

## 3. Results and Discussion

### 3.1. Morphological, Phylogenetic, and ITS2 Secondary Structure Analyses of C. saccharophilum

**Figure 2:**
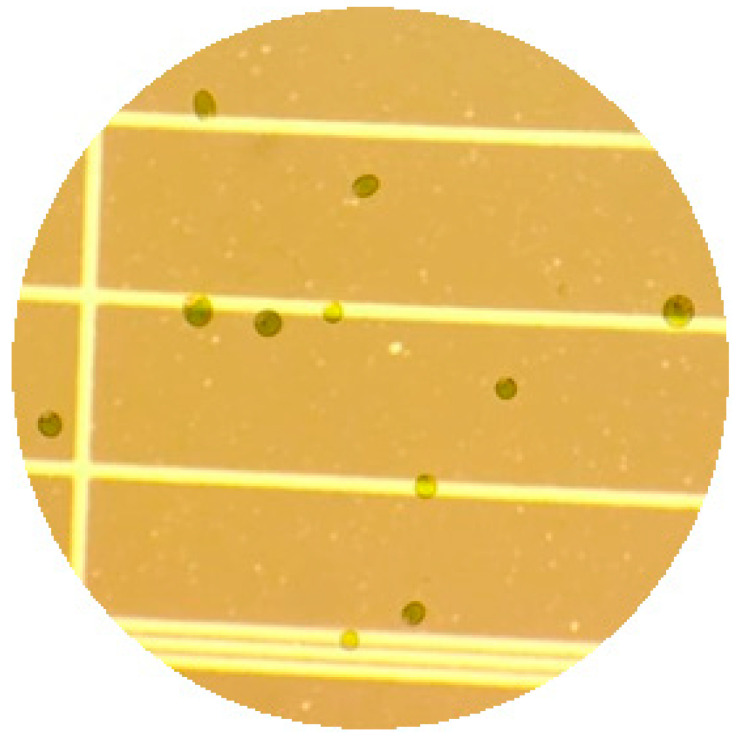
*C. saccharophilum* observed under bright-field microscopy (40×).

**Figure 3:**
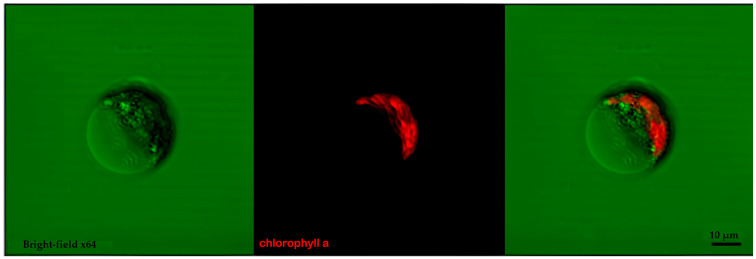
*C. saccharophilum* observed by confocal microscopy 64×. Confocal images were acquired using two configurations: chlorophyll autofluorescence and transmission imaging. Chlorophyll autofluorescence was detected using a 488 nm excitation laser, which induces the characteristic intrinsic fluorescence of chlorophyll pigments. The transmission channel (bright field/DIC) was used to enhance visualization of cellular morphology and structural features. No fluorescent staining was required, as signal detection relied on the natural autofluorescence of the cells. Acquisition parameters were adjusted to optimize signal intensity, contrast, and spatial resolution.

Cells of *C. saccharophilum* were small, coccoid, and uniformly green, measuring 3–5 µm in width and 5–8 µm in length (Figure 2 and Figure 3). No distinctive morphological characteristics were observed that clearly differentiate this strain from other *Chloroidium* taxa. When cells became encysted, they adopted a spherical morphology with previous descriptions in the literature [4].

**Figure 4:**
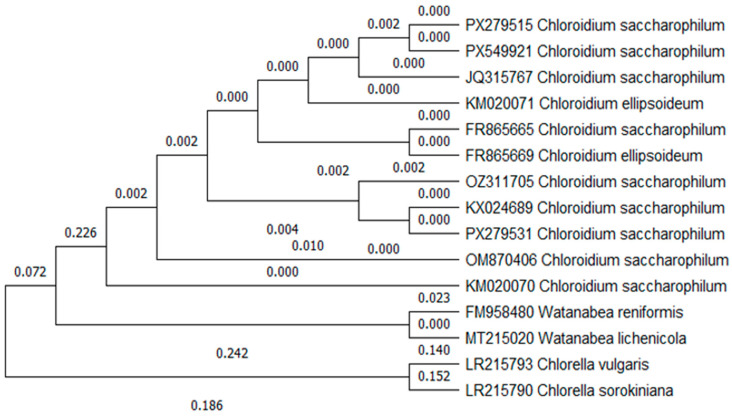
The phylogenetic tree generated with ITS marker sequences (ITS4 and ITS5 rDNA) shows that the studied strain of *C. saccharophilum* (OZ311705) is actually this species. The tree is composed of different sequences of *C. saccharophilum* and its taxonomic position compared to *C. ellipsoideum*, *Watanabea* spp., and *Chlorella* spp. as an outgroup.

The sequence obtained using primers ITS4 and ITS5 was used to perform a BLASTn and phylogenetic tree (Figure 4). The BLASTn analysis yielded a 99.87% identity match with *C. saccharophilum*. The strain of *C. saccharophilum* (OZ311705), isolated from Laguna Blanca (Punta Arenas), is positioned in a well-supported monophyletic clade and grouped with other strains of *C. saccharophilum.* The genetic differences between strains are minimal (0.000–0.002); this confirms that the strain analyzed belongs to the genus *Chloroidium* and validates the species identification. Sequences from the genus *Watanabea* and *Chlorella* were used as an outgroup to confirm and promote directionality to the phylogenetic tree.

**Figure 5:**
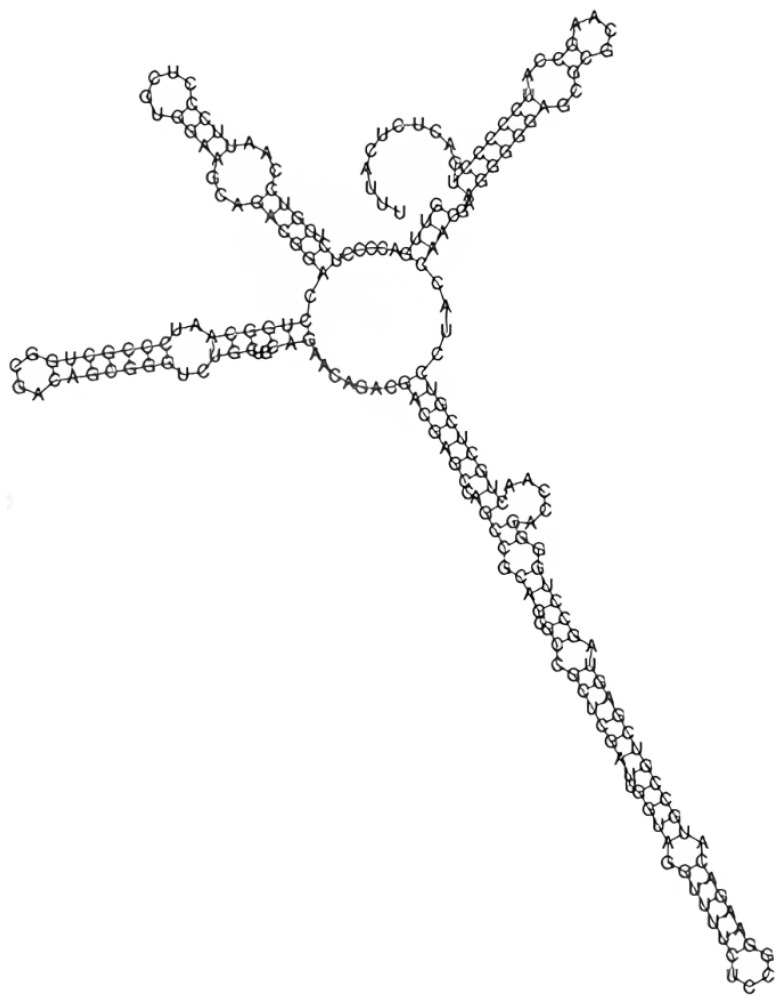
Secondary structure predicted for the ITS2 transcript of strain OZ311705. The bending was based on the minimum free energy criterion (MFE). The free energy of the thermodynamic assembly was −97.16 kcal/mol.

The modeling of the ITS2 secondary structure for the *C. saccharophilum* strain shows a thermodynamically stable conformation, with a free energy of the thermodynamic ensemble of −97.16 kcal/mol. The structure generated by the minimum free energy (MFE) model displayed a topology characteristic of the genus, featuring four main helices radiating from a central loop. Regarding the Boltzmann ensemble analysis, the MFE structure frequency and ensemble diversity were 1.14% and 13.01, respectively. These values indicate that, although a dominant stable structure exists, the transcript possesses inherent plasticity, likely fluctuating between conformational states of similar energy. Consequently, the predicted ITS2 secondary structure of strain OZ311705 (Figure 5) showed no CBCs when it was compared to the reference strain (PX279531), confirming its taxonomic identity.

**Figure 6:**
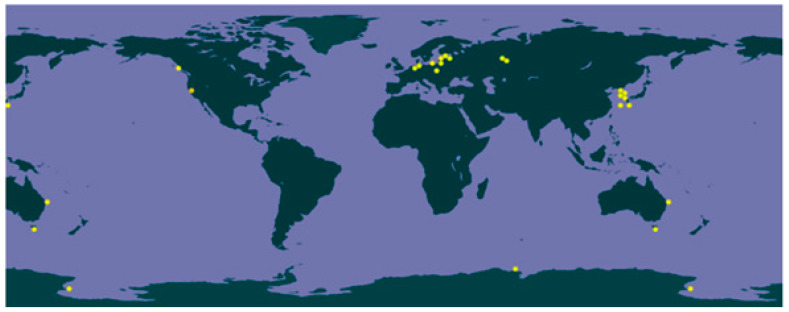
Map of confirmed occurrences of *C. saccharophilum* (W. Krüger); Darienko et al. [4] https://www.gbif.org/species/7356384 (accessed on 14 May 2025).

*C. saccharophilum* was identified in the Laguna Blanca aquifer, representing the only confirmed record of this species in Chile and the sole report from the Southern Cone since 1986 [32]. Figure 6 illustrates the global distribution of *C. saccharophilum,* highlighting its worldwide distribution. However, most data are from polar and cold-climate regions, whereas reports from continental America are scarce.

### 3.2. Comparative Environmental Conditions Between Mars and the Hyper-Arid Atacama Desert

The analogies between the Atacama Desert and Mars are particularly obvious in terms of extreme aridity, intense UV radiation, oxidizing and saline soils, and geomorphological landscapes with strong visual and structural similarities. Nevertheless, it is essential to emphasize the fundamental differences that make Mars an even more hostile environment (Table 3), including ultra-low temperatures, extremely low atmospheric pressure, intense galactic and solar radiation, and the complete absence of wildlife and vegetation.

### 3.3. Performance of C. saccharophilum Under Laboratory Conditions

Laboratory cultures of *C. saccharophilum* were maintained under controlled conditions at temperatures of 16 °C (T1), 19 °C (T2), and 22 °C (T3), with a salinity of 150 g L^−1^. Surface irradiance at the photobioreactor was maintained at 200 µmol m^−2^ s^−1^.

**Figure 7:**
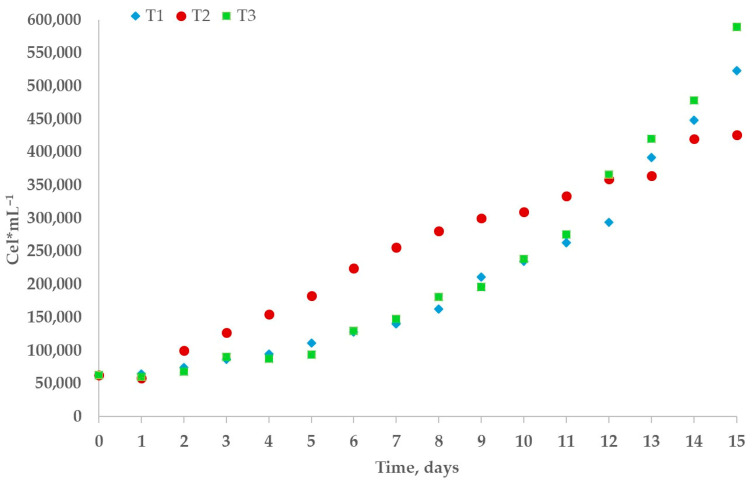
Effect of temperature by treatment on *C. saccharophilum* in cell concentration (CC), with T1 = 16 °C, T2 = 19 °C, and T3 = 22 °C.

An ANOVA revealed a significant effect of temperature on growth performance under indoor conditions (*p* < 0.05). This result was confirmed by a Tukey post hoc test, which indicated that treatment T3 had a statistically significant effect. By day 15, the highest biomass concentration was observed in T3, reaching 0.17 g L^−1^, with a maximum specific growth rate of approximately 0.09 day^−1^ and a maximum productivity of approximately 0.017 g L^−1^ day^−1^ (Figure 7). These values are broadly consistent with those reported by Piasecka and Baier [40], who cultivated *C. saccharophilum* under similar temperature and photon flux conditions (22 °C and 200 µmol m^−2^|s^−1^). In contrast, treatments T1 and T2 showed no significant differences in biomass concentration.

Piasecka and Baier (2022) [40] reported substantially higher growth rates (0.39 day^−1^) and biomass concentrations under favorable autotrophic conditions. The lower biomass concentration observed in the present study may be explained by differences in culture parameters, particularly the use of KSP medium, whereas Piasecka and Baier employed Bold’s basal medium supplemented with 1% (*w*/*v*) molasses. Their study was conducted at lower irradiance (80 µmol m^−2^ s^−1^) and at 20 °C, both factors known to strongly influence microalgal growth [41].

**Figure 8:**
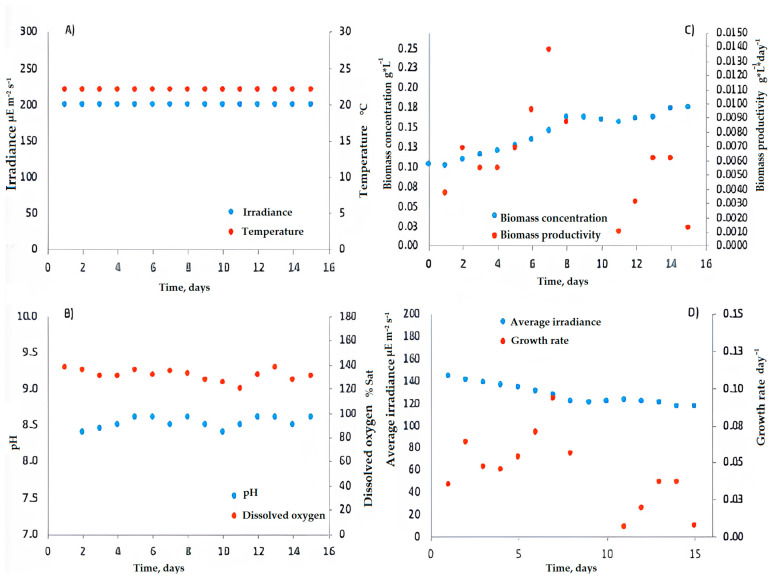
Data from batch culture of *C. saccharophilum* in Laboratory T3. (**A**) Irradiance and Temperature behavior over Time (**B**) pH behavior over Time (**C**) Biomass concentration in grams per Litre and Biomass productivity in grams per Litre over Time and (**D**) Average irradiance and growth rate over time.

Based on these comparisons, it was demonstrated that under indoor laboratory conditions, an optimal irradiance range for *C. saccharophilum* of between 80 and 200 µmol m^−2^ s^−1^ can be obtained. During batch cultivation, average irradiance decreased from 130 to 100 µmol m^−2^ s^−1^ as biomass concentration increased, resulting in a reduction in the specific growth rate from 0.09 to 0.04 day^−1^ (Figure 8). The pH was maintained at 8.5 ± 0.1, while the maximum daily dissolved oxygen concentration reached 137 ± 5% saturation (Figure 8). pH control is crucial to maximize microalgal productivity and prevent cellular damage [41,42,43].

When microalgae are exposed to high salinity, they experience combined osmotic and ionic stress, leading to reduced growth and impaired photosynthetic performance. Under such conditions, metabolic resources are redirected from growth toward the synthesis and storage of lipids, carbohydrates, and protective compounds, explaining the low productivity and growth rates observed in this study [44,45]. Halophytic and halotolerant microalgae represent a physiologically diverse and ecologically relevant group capable of completing their life cycles under high salinity. Well-studied examples include *Dunaliella*, *Picochlorum*, *Nannochloris*/*Nannochloropsis*, *Chloroidium*, and halophilic diatoms [46,47,48].

### 3.4. Performance of C. saccharophilum Under Outdoor Conditions

**Figure 9:**
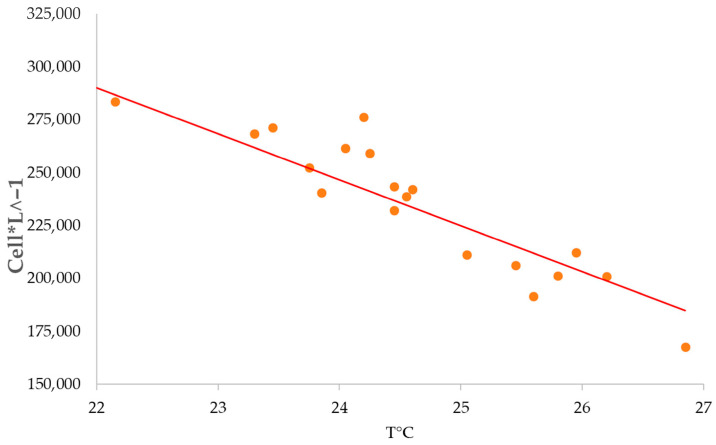
Correlation between cell concentration and temperature in outdoor culture. Cell concentration follows a negative correlation with the increase of temperature, with the lowest concentration before culture failure happening at almost 27°C.

Outdoor microalgae cultivation systems are exposed to daily and seasonal temperature fluctuations that strongly influence growth and productivity. Temperatures exceeding the species-specific optimum are particularly harmful, although their quantitative effects are rarely reported [49,50,51]. Outdoor cultures were settled in the desert conditions at the Canchones Experimental Station (Arturo Prat University), showing a strong negative Spearman correlation (−0.89) between average temperatures above 25 °C and cell concentration (Figure 9). ANOVA analysis (*p* < 0.05) confirmed the significant impact of temperature on cell concentration, decreasing sharply above 25 °C. Minimum temperature did not show a significant effect.

**Figure 10:**
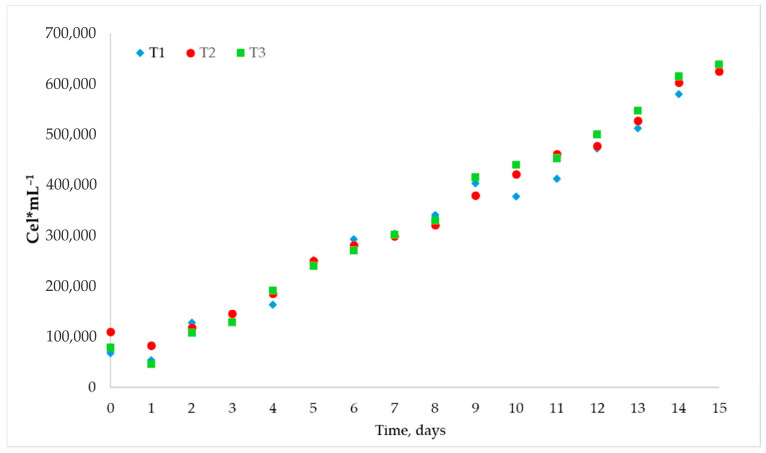
Raceway bioreactors culture *C. saccharophilum* by treatment in the Atacama Desert, T1: 10% nitrate; T2: 15% nitrate; and T3: 0% nitrate added.

At the Pampa del Tamarugal site, cultures were exposed to average solar irradiance ranging from 600 to 2472 µmol m^−2^ s^−1^ along a 15-day period. No significant differences in growth were observed at the three raceway photobioreactors used (Figure 10).

**Figure 11:**
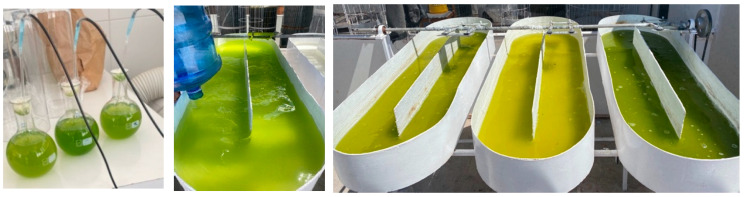
Raceway-type bioreactor system located in the Atacama Desert. Each one was a step during the culture scaling; the color difference in T3 is due to its higher biomass than its counterparts T1 and T2.

**Figure 12:**
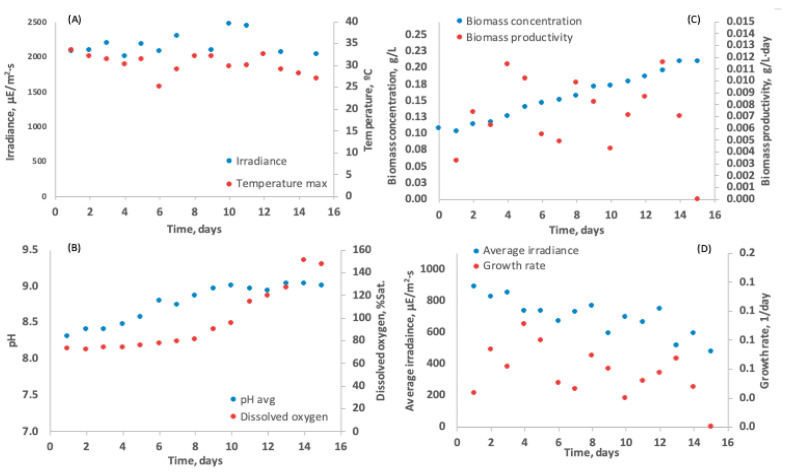
*C. saccharophilum* data from batch culture of in raceway-type bioreactors in the Atacama desert; T3: N 0%. (**A**) Irradiance and maximum Temperature over Time (**B**) pH behavior over Time (**C**) Biomass concentration in grams per Litre and Biomass productivity in grams per Litre per day over Time and (**D**) Average irradiance and growthrate per day over Time.

Within the raceway systems (Figure 11), culture temperatures ranged from 25 to 33 °C, while pH values remained between 8.3 and 9.0 (average 8.6). Dissolved oxygen concentrations increased over time as biomass accumulated, ranging from 73 to 150% saturation. Higher solar irradiance enhanced photosynthetic rates, CO_2_ demand, and oxygen production [52,53]. The maximum specific growth rate reached under outdoor conditions was 0.09 per day^−1^, while maximum productivity was 0.01 g L^−1^ per day^−1^ (Figure 12). Despite the exposure to higher irradiance and temperature, productivity was similar to that obtained in laboratory conditions. This might be due to the high salinity that induces a survival-oriented metabolic state [41,54]. Tolerance to extreme irradiance in *C. saccharophilum* remains poorly studied. However, studies on *C. saccharophilum* under continuous high-light conditions have shown increased photosynthetic capacity accompanied by reduced Chl and carotenoid content, a typical high-light acclimation response [55].

### 3.5. Nitrate and Phosphate Uptake and Oxygen Production

**Figure 13:**
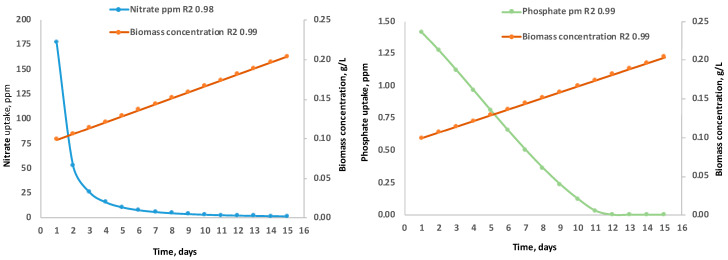
Nitrate and phosphate uptake trends in the cultivation of *C. saccharophilum* in raceway-type bioreactors in the Atacama Desert.

Although there are no previous studies that address *C. saccharophilum* cultivation in raceway systems, research on *Chlorella* spp. provides useful comparative trends. Raceway systems can achieve nitrogen removal efficiencies of 70–100% and phosphorus removal efficiencies of 40–75% under conditions of high biomass and extended residence time [56,57]. In this study, initial nitrogen and phosphorus concentrations were intentionally low to induce nutrient stress. Both nutrients were fully depleted by days 10 to 12. Nitrate uptake followed a linear trend, whereas phosphate uptake followed a power-law function, with both nutrients reaching depletion simultaneously (Figure 13), in contrast to previous reports [58]. Nutrient uptake ratios are species- and condition-dependent, and deviations from canonical patterns are common. In *Chlorella* spp., C/N and N/P ratios vary with irradiance and growth phase [59,60,61].

**Figure 14:**
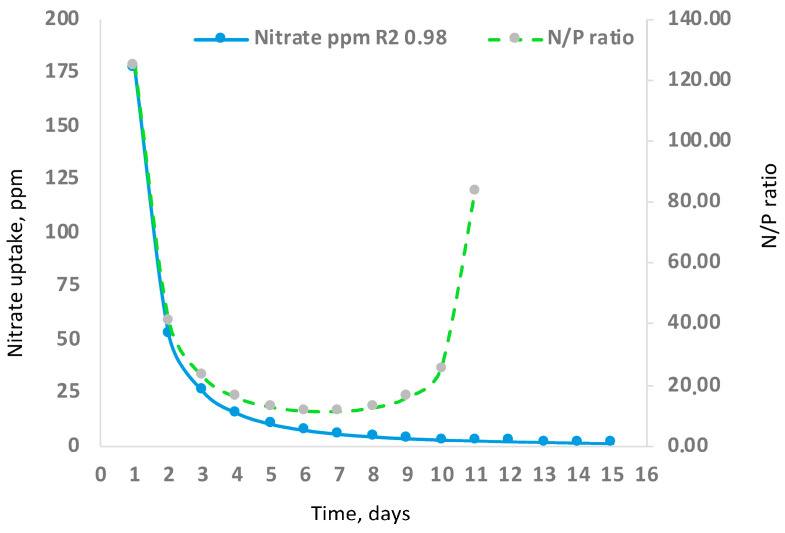
Nitrate uptake trends and N/P ratio in the cultivation of *C. saccharophilum* in raceway-type bioreactors in the Atacama Desert.

The N:P ratio exhibited a concave temporal pattern, reaching a minimum optimal value of 11.8 N:1 P on day 7 (Figure 14). Despite the dependence on atmospheric CO_2_, the biomass production increased at a constant rate. High surface-to-volume ratios in raceway systems facilitate oxygen degassing, particularly at elevated temperatures, preventing excessive oxygen accumulation (>146% saturation) and minimizing photoinhibition [62,63].

### 3.6. Effects of Salinity on Carotenoid Content and Chlorophyll a/b Ratio

**Figure 15:**
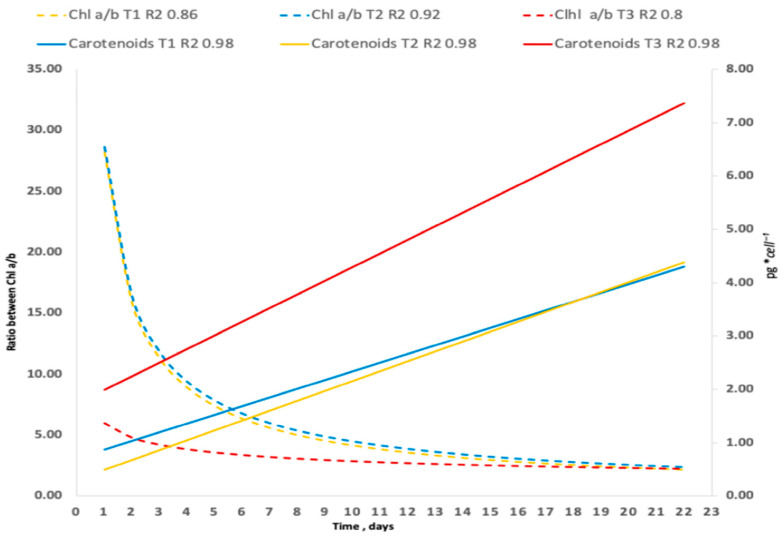
Tendencies of the effect of stress treatment on carotenoid accumulation and ratio between Chl a/b in *C. saccharophilum*.

Salt stress and nutrient limitation are widely used strategies to enhance pigment and lipid production in microalgae [64,65]. Cultures exposed to high salinity (150 g L^−1^), maximum irradiance (2472 µmol m^−2^ s^−1^), nitrogen limitation, and high UV radiation showed significant effects on pigment synthesis (*p* < 0.05). Treatment T3 exhibited the highest carotenoid accumulation, reaching 7.36 pg cell^−1^, equivalent to 3.77 mg L^−1^ (Figure 15). Chl a and b ratios decreased across treatments, reaching a value of 7.36. That indicates that the cells are adapted to intense light stress exposure. Quantitative data on Chl a/b ratios in *C. saccharophilum* remain scarce. In halophytic plants, salinity tolerance is often associated with pigment reorganization and increased synthesis of antioxidant compounds; a similar response was observed in this study [66,67].

High irradiance generally improves photosynthesis rate, CO_2_ uptake, and O_2_ production up to a species-specific optimum. Beyond this threshold, excess light and oxygen induce photoinhibition and photorespiration, respectively. In this study, the increase of oxygen production and carotenoid accumulation indicate the activation of photoprotective mechanisms [53].

The high Chl a/b ratios observed correspond to exposure to intense solar radiation, averaging approximately 2277 µmol m^−2^ s^−1^, according to previous observations [68].

### 3.7. Microalgae and Cyanobacteria Under Simulated Martian Conditions

The research of microalgae and cyanobacteria culturing under simulated Martian conditions has been focused on analyzing their potential relevance for life-detection studies and for supporting future human missions. These microorganisms are interesting due to their photosynthetic capacity, oxygen production, rapid growth rates, and resistance to extreme environments.

Among cyanobacteria, *Chroococcidiopsis* spp. are especially noticeable due to their exceptional resistance to desiccation, UV radiation, temperature extremes, and salinity as well as their ability to grow on Martian regolith analogs and produce oxygen. Other genera, such as *Nostoc* and *Anabaena*, have been investigated for their nitrogen-fixing capacity. Green microalgae, including *Chlorella vulgaris* and *Haematococcus pluvialis*, are useful for their high nutritional content, biomass productivity, and radiation tolerance, making them promising candidates for photobioreactor-based and life-support systems. Although less extensively studied, diatoms have also demonstrated high photosynthetic efficiency under extreme conditions.

Overall, previous studies confirm the remarkable resistance of many microalgae and cyanobacteria to Martian-like stressors, while consistently highlighting that the availability of liquid water remains a critical limiting factor for survival and growth. Table 4 summarizes microalgae and cyanobacteria that have already been tested under simulated Martian conditions.

### 3.8. Implications for C. saccharophilum

Based on the information summarized in Table 4, there is no actual evidence that supports the use of *C. saccharophilum* as a suitable candidate for survival or growth under Martian climatic conditions. Furthermore, no studies were found that directly compare the physiological similarities between *C. saccharophilum* and *Dunaliella salina*. Nevertheless, *Dunaliella salina* is a halophilic marine microalga widely recognized for its ability to produce bioactive compounds such as carotenoids and terpenes, with applications in the food and pharmaceutical industries as well as in drug-delivery systems and gene therapies [74,75]. In the light of the results presented in this study, further research focused on Saccharophilum is required to better assess its physiological limits and potential relevance in extreme and Mars-analog environments.

The application of genetic engineering is a powerful strategy to adapt this strain to Martian environmental conditions. Targeted genetic modifications could enhance specific traits such as cold tolerance, DNA repair capacity, photoprotection, and efficient CO_2_ utilization while optimizing metabolic pathways related to oxygen production, biopolymer synthesis, or nutritional biomass generation [76,77]. However, many stress-tolerance traits are polygenic and involve complex regulatory networks, making them difficult to improve solely through single-gene editing approaches [78,79,80].

An alternative or complementary strategy is Adaptive Laboratory Evolution (ALE), which involves the cultivation of microalgae over multiple generations under controlled stress conditions (e.g., low temperature, high salinity, nutrient limitation, or toxic compounds) to select the optimal one for beneficial mutations. This approach can yield strains with enhanced thermotolerance, improved growth performance, higher photosynthetic efficiency, and increased resistance to chemical and environmental stressors, thereby progressively conditioning them to Mars-like conditions [2,77,78,79,80,81,82]. Unlike targeted genetic engineering, ALE operates at the whole-genome level, enabling the emergence of robust phenotypes without prior knowledge of the specific genes involved. Moreover, ALE-derived strains are often considered non-genetically modified organisms (non-GMO) under many regulatory frameworks [77,78,83].

## 4. Conclusions

This study provides a comprehensive physiological, molecular, and ecological characterization of a *C. saccharophilum* strain isolated from the Laguna Blanca aquifer in southern Chile, constituting the first confirmed record of this species in the Southern Cone. Phylogenetic analyses based on ITS rDNA sequencing and ITS2 secondary structure supported its taxonomic identity and revealed minimal genetic divergence from previously described strains, reinforcing its worldwide sparsely reported distribution. Under laboratory conditions, *C. saccharophilum* showed optimal growth at moderate temperatures and a pronounced tolerance to high salinity, confirming its halotolerant nature. Outdoor cultivation under hyper-arid Atacama Desert conditions demonstrated that higher temperatures above the optimal range significantly constrained biomass accumulation, while high irradiance and nutrient limitation triggered photoprotective responses, including carotenoid synthesis. However, pigment accumulation remained comparatively low, even under combined stress conditions, indicating a conservative metabolic strategy oriented toward survival rather than secondary metabolite overproduction.

The observed physiological traits suggest that *C. saccharophilum* is well-adapted to extreme saline and irradiance-stressed environments, making it a promising candidate for industrial applications such as saline bioremediation and biomass production. Nonetheless, its limited carotenoid yield highlights the need for further optimization, potentially through multi-stress approaches involving ultraviolet radiation, metal exposure, or refined nutrient regimes. Future research should also explore its long-term performance under controlled extreme conditions and its relevance as a biological model for Mars-analog environments, thereby expanding both its applied and astro-biological significance.

## Figures and Tables

**Table 1:** Culture medium for experimental growth of *C. saccharophilum* in the laboratory.

KSP Medium/Composition	g L^−1^	Source
KNO_3_ agricultural origin	0.5	Chemical and Mining Society of Chile (SQM), Santiago, Chile
Sea salt	150	Mohican-Sodimac, Santiago, Chile
Triple superphosphate	0.05	Fertilizin Best Garden, Santiago, Chile
Sol. EDTA-Fe (mL L^−1^)	0.1	Oregon CHEM GROUP, Santiago, Chile

**Table 2:** Treatments for temperature measurement.

REP	T1	T2	T3
1	16 °C 1	19 °C 1	22 °C 1
2	16 °C 2	19 °C 2	22 °C 2
3	16 °C 3	19 °C 3	22 °C 3

The thermostat uncertainty was ±1 °C, with a standard deviation of 2σ.

**Table 3:** Comparison of environmental parameters between Mars (today) and the hyper-arid Atacama Desert.

Aspect	Mars (Today)	Hyper-Arid Atacama	Citations
Atmospheric pressure	~6–8 mbar	~1013 mbar (terrestrial)	(Martínez et al., 2017) [33]; (Ritter et al., 2019) [34]
Liquid precipitation	None	<2 mm year^−1^, episodic	(Ritter et al., 2019) [34]; (Arens et al., 2024) [35]
Typical temperature range	~–125 to ~20 °C	~–6 to ~38 °C	(Martínez et al., 2017) [33]; (Mckay et al., 2003) [36]
Water availability	Ice/frost; possible transient brines	Extremely scarce rainfall, fog, localized subsurface moisture	(Mckay et al., 2003) [36]; (Azua-Bustos et al., 2015) [37]; (Ritter et al., 2019) [34]; (Shen et al., 2021) [38]
UV radiation	Very high (thin atmosphere)	Very high (clear sky, altitude, ozone depletion)	(Shen et al., 2021) [38] (Azua-Bustos et al., 2022) [39];

**Table 4:** Microalgae and cyanobacteria evaluated under Mars-like conditions.

Microalgae/ Cyanobacterium	Key Trait Relevant to Mars	Evidence Under Mars-like Conditions	Citations
*Chlorella vulgaris*	High tolerance to elevated CO_2_ and low pressure; efficient photosynthesis	Grows and produces O_2_ under simulated Martian atmospheres; shows higher performance than under terrestrial air	Likai et al., 2025 [69]; Cycil et al., 2021 [70]; Mapstone et al., 2022 [71]; Macário et al., 2022 [72]
*Dunaliella salina*	Halophilic; tolerant to high salinity and osmotic stress	Sustains biomass production at 160 ± 20 mbar (low-pressure conditions)	Cycil et al., 2021 [70];
*Chloromonas brevispina*	Adapted to cold environments, high radiation, and naturally low pressure	Exhibits robust growth at pressures ranging from 330 to 80 mbar	Cycil et al., 2021 [70];
*Spirulina/Arthrospira platensis*	Edible cyanobacterium with high protein content	Grows using a mixture of Martian regolith simulant and human urine under Mars-like atmospheric conditions	Cycil et al., 2021 [70]; Macário et al., 2022 [72]; Fais et al., 2022 [73]
*Synechococcus nidulans*	Extremophilic, potentially edible cyanobacterium	Capable of growth in media supplied with Martian CO_2_ and simulated regolith	Cycil et al., 2021 [70]
*Nostoc muscorum*	Potentially edible cyanobacterium	Grows using only water and MGS-1 regolith simulant under terrestrial atmospheric conditions	Cycil et al., 2021 [70]

## Data Availability

The original contributions presented in this study are included in the article. Further inquiries can be directed to the corresponding author(s).

## Abbreviations

The following abbreviations are used in this manuscript:

PB	Biomass productivity in g*L^−1^*day^−1^
CBf	Final biomass concentration in g*L^−1^
CB0	Initial biomass concentration in g*L^−1^
tf:	Final time in days
t0	Initial time in days
μ	Growth rate in day^−1^
CC	Cell concentration in Cel*mL^−1^
CBn	Biomass concentration on a specific day in g*L^−1^
Chl	Chlorophyll
Ax	Absorbance at a specific wavelength, with x representing the wavelength.
